# Mesenchymal stem cells and secretome as modulators of neuroinflammation in neurological disorders

**DOI:** 10.1186/s12967-026-08052-x

**Published:** 2026-03-25

**Authors:** Dabao Yao, Luwei Nie, Xia Liu, Xuan Wu, Yingxin Tang, Chao Pan, Shiling Chen, Danyang Chen, Dongcheng Wu, Hao Nie, Na Liu, Zhouping Tang

**Affiliations:** 1 Department of Neurology, Tongji Hospital, Tongji Medical College, Huazhong University of Science and Technology, No. 1095, Jiefang Avenue, Qiaokou District, Wuhan, Hubei 430030 China; 2 Department of Neurology, Jingzhou Hospital, Yangtze University, Jingzhou, China; 3 Department of Biochemistry and Molecular Biology, Wuhan University School of Basic Medical Sciences, Wuhan, Hubei 430030 China; 4 Wuhan Hamilton Biotechnology Co., Ltd., Wuhan, Hubei 430030 China; 5 Department of Geriatrics, Tongji Hospital, Tongji Medical College, Huazhong University of Science and Technology, Wuhan, Hubei China

**Keywords:** Mesenchymal stem cells, Secretome, Neuroinflammation, Immunomodulation, Neurological disorders

## Abstract

**Background:**

Neuroinflammation is a critical pathogenic driver in a wide spectrum of neurological disorders, contributing to significant morbidity and presenting a formidable therapeutic challenge. Among emerging regenerative approaches, mesenchymal stem cells (MSCs) have garnered significant attention for their potent capacity to modulate this detrimental immune response, offering hope for conditions ranging from acute brain injury to chronic neurodegeneration.

**Objective:**

This review aims to comprehensively synthesize the current understanding of how MSCs and their secretome, particularly extracellular vesicles (EVs), therapeutically modulate neuroinflammation. We seek to elucidate the key molecular and cellular mechanisms of action and to critically evaluate the evidence for these therapies across various neurological disease models.

**Evidence review:**

This review synthesizes the evolving literature on MSC-mediated immunomodulation, highlighting the therapeutic transition from cell replacement to secretome-based strategies. We examine pivotal studies elucidating the molecular mechanisms by which MSCs and their secretome regulate glial phenotypes and inflammatory pathways, preserve blood-brain barrier integrity, and modulate peripheral immune responses. Furthermore, we critically analyze therapeutic efficacy across preclinical models of acute and chronic neurological disorders and assess the current status of clinical translation.

**Findings:**

The primary therapeutic action of MSCs is mediated by their paracrine secretome, not cell replacement. Key findings demonstrate that MSC-derived EVs deliver bioactive cargo (e.g., microRNAs, TSG-6) that actively reprograms microglia and astrocytes from a pro-inflammatory to a neuroprotective phenotype and suppresses critical inflammatory signaling pathways, such as TLR4/NF-κB and the NLRP3 inflammasome, thereby reducing neuronal damage, preserving blood-brain barrier integrity, and fostering an environment conducive to endogenous repair.

**Conclusion:**

MSCs and their cell-free secretome represent a promising therapeutic platform for neurological disorders by directly targeting neuroinflammation. While clinical translation is advancing, significant challenges in standardization, manufacturing, and regulatory approval must be addressed. Future progress will depend on developing next-generation, potentially bioengineered, secretome-based products with defined potency to bring this regenerative strategy from the laboratory to the clinic.

## Introduction

Neurological disorders, a vast and heterogeneous group of conditions affecting the central and peripheral nervous systems, impose a profound and escalating burden on global health and society. These conditions range from acute insults such as ischemic stroke, traumatic brain injury (TBI), and spinal cord injury (SCI) to chronic, progressive neurodegenerative diseases including Alzheimer’s disease (AD), Parkinson’s disease (PD), multiple sclerosis (MS), and amyotrophic lateral sclerosis (ALS). While the specific etiologies of neurological disorders are diverse, a converging body of evidence points to neuroinflammation as a critical common pathogenic factor [1, 2]. Neuroinflammation, the inflammatory response within the central nervous system (CNS), exhibits a dual nature. While acute inflammation is a necessary protective response to injury, chronic and dysregulated neuroinflammation becomes maladaptive, contributing directly to neuronal damage, synaptic dysfunction, and the progressive nature of many of these diseases [3, 4]. Neuroinflammation involves the activation of resident CNS immune cells, such as microglia and astrocytes, and can be exacerbated by the infiltration of peripheral immune cells, creating a self-perpetuating cycle of damage [5, 6]. Recognizing neuroinflammation as a converging hub in the pathogenesis of a wide spectrum of neurological disorders has made it a prime target for therapeutic intervention.

In the quest for effective treatments for neurological disorders, cell-based therapies have emerged as a highly promising frontier. Among various cell types, mesenchymal stem cells (MSCs), also referred to as mesenchymal stromal cells, have garnered significant attention due to their unique therapeutic properties. MSCs are multipotent stromal cells that can be sourced from diverse tissues, including bone marrow, adipose tissue, umbilical cord, and dental pulp. The therapeutic value of MSCs lies not only in their capacity to self-renew and differentiate into various cell lineages but also in their potent immunomodulatory and regenerative capabilities [7, 8]. MSCs can migrate to sites of injury and inflammation to orchestrate a complex reparative response that includes promoting neurogenesis and angiogenesis, reducing apoptosis, and critically, modulating the neuroinflammatory microenvironment [9, 10]. Immunomodulation represents the central mechanism of MSCs’ therapeutic benefit, allowing these cells to suppress excessive inflammatory reactions that cause secondary damage in conditions like TBI, stroke, and SCI [7, 11]. By simultaneously targeting multiple pathogenic mechanisms—most notably neuroinflammation, the central driver of pathology—MSCs offer a multifaceted approach [12]. Preclinical studies across a wide array of animal models for neurological disorders have demonstrated that MSC transplantation can lead to significant improvements in functional recovery, reduction in lesion volume, and mitigation of pathological hallmarks [13, 14].

The initial rationale for using stem cells in regenerative medicine was centered on the concept of cell replacement—transplanted cells would differentiate into new neurons or glial cells to replace those lost to disease or injury. However, a consensus has emerged from decades of research that the primary therapeutic benefits of MSCs are likely not derived from direct cell engraftment and differentiation, but rather from their powerful paracrine effects [15]. Specifically, by releasing a complex secretome of bioactive molecules—including soluble factors (cytokines, chemokines, growth factors) and extracellular vesicles (EVs)—MSCs modulate the host microenvironment, reducing inflammation, protecting existing cells from apoptosis, and stimulating endogenous repair mechanisms [16, 17].

Within the secretome, EVs—and particularly a subtype called exosomes—have been identified as the key mediators of MSCs’ therapeutic actions. Exosomes are nano-sized vesicles (30–150 nm) that function as natural carriers for intercellular communication, transporting a diverse cargo of proteins, lipids, and nucleic acids, including microRNAs (miRNAs) and other non-coding RNAs [18, 19]. A crucial advantage of exosomes is their ability to cross biological barriers, including the blood-brain barrier (BBB), allowing them to deliver their therapeutic payload directly to injured sites within the CNS [20]. This “cell-free” approach, using either the complete MSC-conditioned medium (MSC-CM) or purified exosomes, offers several advantages over whole-cell therapy. This strategy circumvents risks associated with live cell transplantation, such as immune rejection, tumorigenicity, and vascular occlusion, while offering better safety, stability, and potential for standardization and large-scale production [4, 21, 22].

The recognition that neuroinflammation is a central driver of pathology in a wide range of neurological disorders, coupled with the understanding that MSCs exert their therapeutic effects primarily by modulating this process via their secretome, has created a new therapeutic paradigm. This review aims to provide a comprehensive synthesis of the current state of knowledge on MSC-based therapies, with a specific focus on their mechanisms of neuroinflammation modulation. We first discuss the central role of neuroinflammation as a common pathogenic mechanism in a variety of CNS conditions. Subsequently, we explore the immunomodulatory mechanisms of MSCs and their secretome on key cellular players in the CNS. We then survey the preclinical and clinical evidence for MSC-based therapies across a spectrum of neurodegenerative diseases and acute CNS injuries. Finally, we address the critical aspects of clinical translation, summarizing progress from clinical trials and discussing the significant challenges and regulatory considerations that must be navigated to bring these promising therapies from the laboratory to the clinic.

## Pathophysiological role of neuroinflammation in neurological disorders

**Fig. 1:**
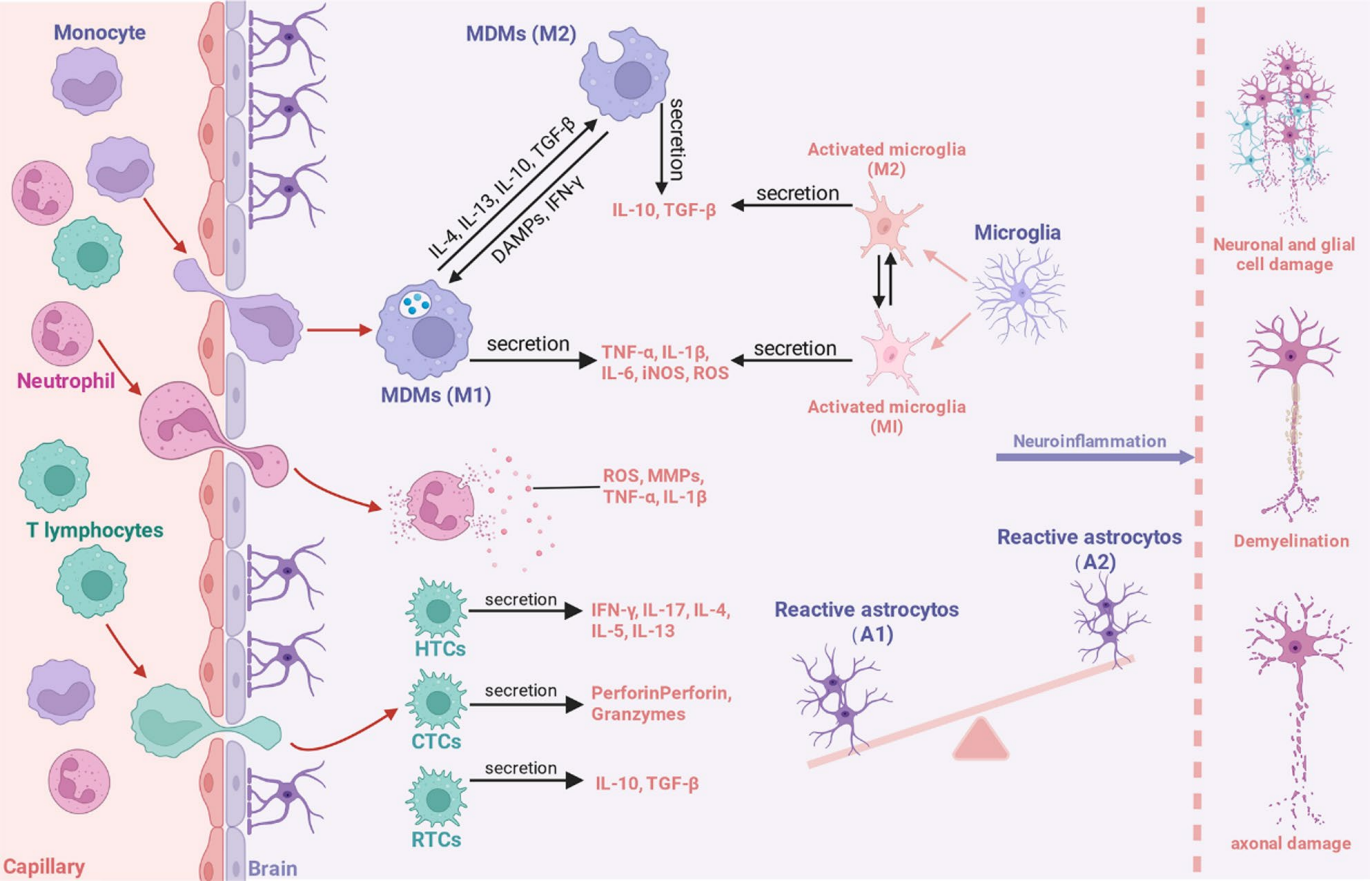
The cellular cascade of neuroinflammation in central nervous system disorders. Disruption of the blood-brain barrier allows peripheral immune cells (monocytes, neutrophils, T lymphocytes) to infiltrate the central nervous system (CNS). Monocytes differentiate into monocyte-derived macrophages, which polarize into either a pro-inflammatory M1 phenotype, secreting cytotoxic factors like TNF-α, IL-1β, IL-6, and reactive oxygen species, or an anti-inflammatory M2 phenotype, which secretes reparative cytokines such as IL-10 and TGF-β. T lymphocytes differentiate into various effector subsets: pro-inflammatory T helper cells releasing cytokines like IFN-γ and IL-17, cytotoxic T cells releasing perforin and granzymes, and immunosuppressive regulatory T cells that secrete IL-10 and TGF-β. Microglia, the CNS-resident immune cells, become activated and also polarize towards either a neurotoxic M1 or a neuroprotective M2 state. Similarly, astrocytes become reactive, adopting a neurotoxic A1 phenotype, which dominates over the neuroprotective A2 phenotype, tipping the balance towards pathology. This sustained and imbalanced neuroinflammatory state, driven by both peripheral and central cells, culminates in significant neuropathology, including neuronal and glial cell damage, demyelination of axons, and axonal degeneration. MDMs, monocyte-derived macrophages; IL, interleukin; TGF-β, transforming growth factor-β; DAMPs, damage-associated molecular patterns; IFN-γ, interferon-γ; TNF-α, tumor necrosis factor-α; iNOS, inducible nitric oxide synthase; ROS, reactive oxygen species; MMPs, matrix metalloproteinases; HTCs, T helper cells; CTCs, cytotoxic T cells; RTCs, regulatory T cells. Figure created with BioRender.com

Neuroinflammation is a complex, double-edged sword in the CNS. While acute inflammation is a vital protective response aimed at clearing pathogens, removing cellular debris, and initiating tissue repair, its dysregulation or chronic persistence transforms this response into a primary driver of pathology in a vast array of neurological disorders [4]. This detrimental inflammatory state is not merely a consequence of neuronal injury but actively contributes to a self-perpetuating cycle of neurodegeneration [7]. The state is characterized by the sustained activation of resident glial cells, disruption of the BBB, infiltration of peripheral immune cells, and the overproduction of cytotoxic mediators, including pro-inflammatory cytokines, chemokines, reactive oxygen species (ROS), and nitric oxide [23, 24]. This hostile microenvironment undermines neuronal survival, impairs synaptic function, inhibits endogenous regenerative processes like neurogenesis and myelination, and ultimately accelerates disease progression across conditions ranging from acute TBI and stroke to chronic diseases like AD, PD, and MS [6, 25, 26] (Fig. 1). Understanding the key cellular and molecular players in this process is fundamental to developing effective therapeutic interventions.

### The dual role of glial cells

Glial cells, once considered mere structural support for neurons, are now recognized as dynamic and critical regulators of CNS homeostasis and key orchestrators of the neuroinflammatory response [27]. Microglia and astrocytes, the two principal glial cell types, exhibit remarkable plasticity, capable of adopting phenotypes that can be either neuroprotective or neurotoxic depending on the context and nature of the activating stimulus [28]. This dual functionality is central to the balance between physiological repair and pathological damage in the brain.

Microglia, the resident immune cells of the CNS, act as sentinels, constantly surveying their microenvironment for signs of injury or infection. Upon detecting damage-associated molecular patterns (DAMPs) released from injured cells or pathogen-associated molecular patterns, they become activated [6]. This microglial activation is not a monolithic response but rather a spectrum of functional states, often simplified into two opposing poles: the classical M1 (pro-inflammatory) phenotype and the alternative M2 (anti-inflammatory/pro-reparative) phenotype [29]. However, it is increasingly recognized that this M1/M2 dichotomy is an oversimplification, particularly in chronic neurodegeneration. Recent single-cell RNA sequencing studies have identified unique transcriptional states, such as “disease-associated microglia” (DAM), which are characterized by the upregulation of lipid metabolism and phagocytic genes (e.g., *TREM2*, *APOE*) and the downregulation of homeostatic markers (e.g., *P2RY12*) [30–32]. M1-polarized microglia release a barrage of pro-inflammatory cytokines such as tumor necrosis factor-α (TNF-α), interleukin-1β (IL-1β), and interleukin-6 (IL-6), as well as ROS and nitric oxide via inducible nitric oxide synthase (iNOS) [33, 34]. This M1 state, while important for clearing debris and pathogens, becomes highly neurotoxic when sustained, directly contributing to neuronal apoptosis and exacerbating secondary injury in conditions like ischemic stroke, TBI, and SCI [35–37]. In contrast, M2-polarized microglia secrete anti-inflammatory cytokines like interleukin-10 (IL-10) and transforming growth factor-β (TGF-β), along with neurotrophic factors, promoting tissue repair, phagocytosis of cellular debris, and resolution of inflammation [38, 39]. The M1/M2 balance is therefore a critical determinant of disease outcome, and a persistent M1 dominance is a hallmark of chronic neuroinflammation in diseases like AD, PD, and ALS [40–42].

Astrocytes, the most abundant glial cells in the CNS, perform essential homeostatic functions, including maintaining BBB integrity, regulating synaptic transmission, and providing metabolic support to neurons [43]. In response to injury or inflammation, they undergo a process known as reactive astrogliosis. Similar to microglia, reactive astrocytes can adopt distinct phenotypes, broadly categorized as A1 (neurotoxic) and A2 (neuroprotective) [44]. A1 astrocytes, which are induced by factors released from M1 microglia such as IL-1α, TNF-α, and complement component 1q, lose their ability to support neuronal survival and synaptogenesis and instead actively kill neurons and oligodendrocytes [45]. This neurotoxic A1 phenotype has been implicated in the pathology of numerous neurodegenerative diseases, including PD, AD, Huntington’s disease (HD), and ALS [46]. Conversely, A2 astrocytes are induced by stimuli like ischemia and promote neuronal survival and tissue repair. They upregulate the expression of neurotrophic factors and contribute to the resolution of inflammation. However, in chronic injury settings like SCI, reactive astrocytes also contribute to the formation of a glial scar, a dense physical and chemical barrier that, while initially neuroprotective by containing the area of injury, ultimately impedes axonal regeneration [47]. The dynamic interplay and crosstalk between microglia and astrocytes are therefore crucial in shaping the neuroinflammatory microenvironment and determining the fate of neural tissue following injury.

### Peripheral immune infiltration and BBB disruption

The CNS is traditionally considered an immune-privileged site, shielded from the systemic circulation by the BBB. The BBB is a highly selective, semipermeable border of endothelial cells that are connected by tight junctions and surrounded by pericytes and astrocytic end-feet, meticulously controlling the passage of molecules and cells into the brain parenchyma. Neuroinflammation profoundly compromises this critical barrier. Pro-inflammatory cytokines and matrix metalloproteinases (MMPs), released by activated microglia and astrocytes, degrade tight junction proteins (e.g., ZO-1, occludin, claudin-5) and upregulate adhesion molecules (e.g., ICAM-1, VCAM-1) on the surface of endothelial cells [48, 49]. This process of BBB disruption increases its permeability, leading to vasogenic edema and, critically, facilitating the infiltration of peripheral immune cells into the CNS.

Once the barrier is breached, a secondary wave of inflammation ensues as peripheral leukocytes, including neutrophils, monocytes/macrophages, and T lymphocytes, enter the brain tissue [50]. Neutrophils are often among the first responders in acute injuries like TBI and stroke, where they can release cytotoxic enzymes and ROS, contributing to further tissue damage. A specific mechanism of neutrophil-mediated injury is the formation of neutrophil extracellular traps (NETs), web-like structures of DNA and proteins that can exacerbate inflammation and neuronal death [51]. Infiltrating monocytes differentiate into macrophages that, much like microglia, can adopt pro-inflammatory M1 or anti-inflammatory M2 phenotypes, further shaping the local immune landscape [52].

T lymphocytes also play a significant role, particularly in chronic neuroinflammatory and autoimmune diseases like MS, but also in acute injuries and neurodegeneration [53–55]. For instance, the balance between pro-inflammatory T helper 17 (Th17) cells, which secrete IL-17, and immunosuppressive regulatory T cells (Tregs) is often dysregulated. An imbalance favoring Th17 cells over Tregs is observed in acute ischemic stroke and contributes to worse outcomes, while a similar Treg/Th17 imbalance is implicated in TBI [55, 56]. This infiltration of peripheral immune cells establishes a vicious feedback loop, as these cells release additional inflammatory mediators that further activate resident glial cells and perpetuate BBB breakdown, locking the CNS in a state of chronic, damaging inflammation [57].

### Common inflammatory pathways

The coordinated response of glial and immune cells during neuroinflammation is governed by a set of conserved intracellular signaling pathways that translate danger signals into a pro-inflammatory gene expression program. Among the most critical are Toll-like receptor (TLR) signaling, the nuclear factor-kappa B (*NF-κB*) pathway, and the NLRP3 inflammasome [58–60].

TLRs are pattern recognition receptors that recognize pathogen-associated molecular patterns from microbes and DAMPs from damaged host cells [61]. TLR4, for example, is a key receptor for lipopolysaccharide (LPS), a bacterial endotoxin widely used to model neuroinflammation experimentally. Upon activation, TLRs recruit adaptor proteins like myeloid differentiation primary response gene 88 (MyD88), initiating a downstream cascade that culminates in the activation of transcription factors, most notably NF-κB [62].

The NF-κB pathway is a master regulator of inflammation. In resting cells, NF-κB proteins are held inactive in the cytoplasm. Upon stimulation via TLRs or pro-inflammatory cytokines like TNF-α and IL-1β, this inhibition is removed, allowing NF-κB to translocate to the nucleus [50]. There, it orchestrates the transcription of a wide array of pro-inflammatory genes, including those encoding cytokines (TNF-α, IL-1β, IL-6), chemokines, and enzymes like iNOS and cyclooxygenase-2, thereby amplifying and sustaining the inflammatory response [56, 63]. Dysregulation of the NF-κB pathway is a common feature in many neurological disorders, from stroke to AD [64].

The NLRP3 inflammasome is a multiprotein complex within the cytoplasm that plays a central role in activating the potent pro-inflammatory cytokines IL-1β and IL-18 [50]. Its activation is a two-step process: a “priming” signal, often from TLR/NF-κB activation, upregulates the expression of NLRP3 and pro-IL-1β. A second “activation” signal, which can be a variety of stimuli including ROS, mitochondrial dysfunction, or protein aggregates like amyloid-β (Aβ), triggers the assembly of the NLRP3 inflammasome complex [65]. This complex activates caspase-1, an enzyme that cleaves pro-IL-1β and pro-IL-18 into their mature, active forms for secretion. Caspase-1 also cleaves gasdermin D (GSDMD), leading to a form of inflammatory cell death known as pyroptosis [66]. The NLRP3 inflammasome has been strongly implicated in the pathology of TBI, SCI, stroke, AD, and MS, making it a key therapeutic target [67]. Together, these interconnected pathways form a core engine of neuroinflammation, driving the cellular and molecular events that underpin neuronal damage in a wide range of CNS disorders [68, 69].

## The biology of MSCs and secretome

**Fig. 2:**
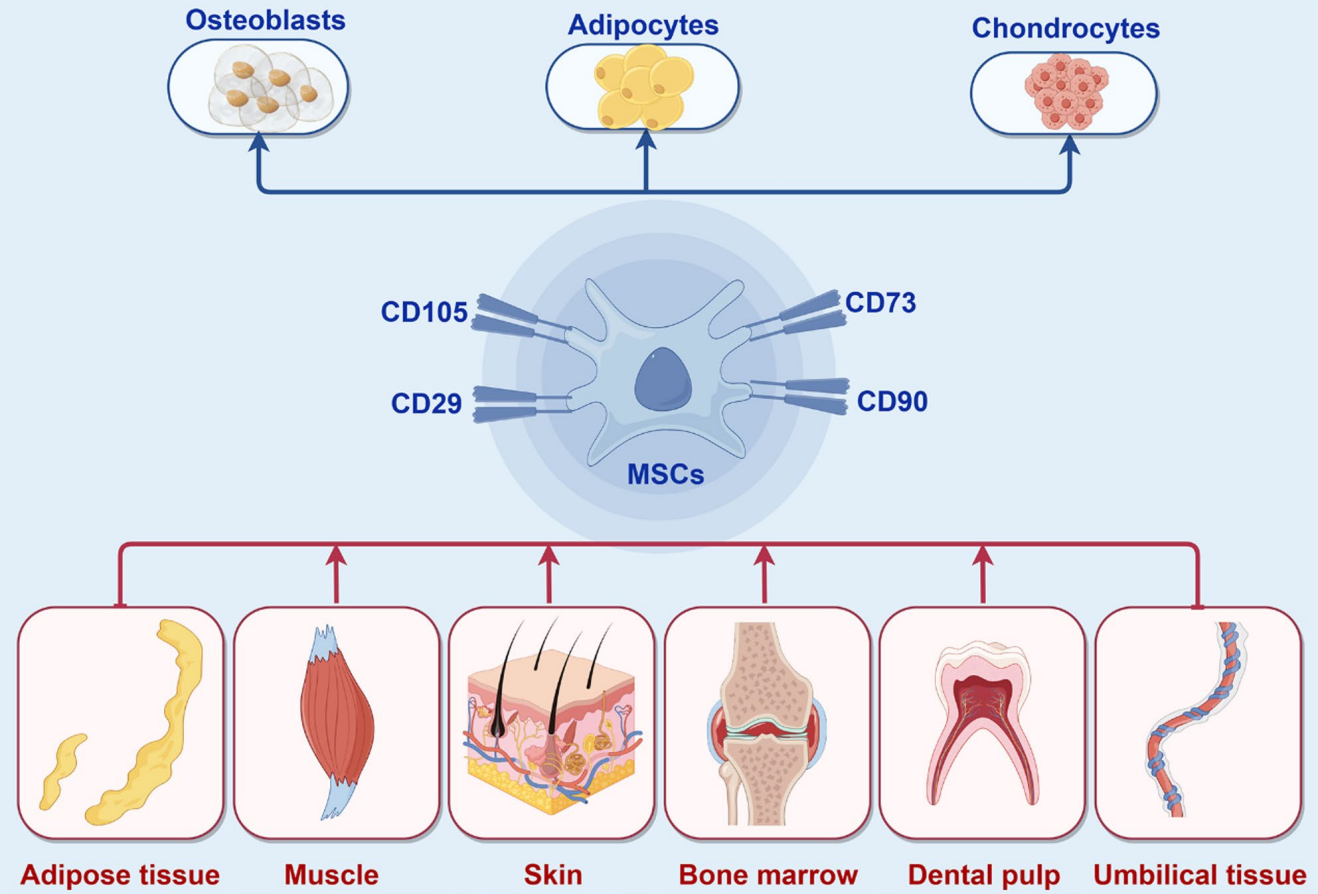
The fundamental identity and therapeutic versatility of mesenchymal stem cells (MSCs). This figure illustrates the dual identity of MSCs that underpins their therapeutic potential. (Top panel) The upper panel demonstrates their defining functional characteristic: multipotency, or the capacity to differentiate into mesodermal lineages including osteoblasts, adipocytes, and chondrocytes. This property formed the initial basis for their use in regenerative medicine. (Center panel) The central panel highlights the key positive surface markers (CD105, CD90, CD73, CD29) used to identify and isolate MSCs, ensuring standardization for research and clinical applications. (Bottom panel) The lower panel showcases their therapeutic versatility, stemming from their presence in a wide range of accessible tissues such as bone marrow, adipose tissue, and perinatal sources like the umbilical cord. The choice of tissue source can influence the cells’ proliferative and immunomodulatory properties, making it a critical consideration for developing targeted therapies for specific neurological disorders. MSCs, mesenchymal stem cells; CD, cluster of differentiation. Figure created with BioRender.com

MSCs are multipotent adult stem cells that have become a focal point of regenerative medicine due to their unique biological properties. As in Fig. 2, MSCs are functionally defined by two core biological properties established by the International Society for Cellular Therapy: their expression of a specific panel of surface markers (e.g., CD73, CD90, CD105) while lacking hematopoietic markers (such as CD45, CD34), and their multipotent capacity to differentiate into mesodermal lineages such as osteoblasts, adipocytes, and chondrocytes [70]. Crucially for their clinical translation, MSCs are not limited to a single source; they can be isolated from a diverse array of tissues including bone marrow, adipose tissue, and perinatal sources like the umbilical cord, each presenting unique profiles of accessibility, proliferation, and immunomodulatory potential [71]. Beyond these defining characteristics, their therapeutic potential in neurological diseases stems largely from their potent secretome, which mediates immunomodulatory, anti-inflammatory, and neurotrophic effects [72]. Understanding the diverse sources from which MSCs can be obtained and the composition of their secretome is crucial for optimizing their therapeutic application.

### Diverse sources of MSCs for neurological applications

MSCs can be isolated from a wide variety of adult and perinatal tissues, each with distinct advantages and characteristics that may influence their therapeutic efficacy. Bone marrow was the first source from which MSCs were identified and remains one of the most extensively studied [73]. Bone marrow mesenchymal stem cells (BM-MSCs) have been used in numerous preclinical models of neurological disease, demonstrating effects such as reducing microglial pyroptosis in intracerebral hemorrhage (ICH) and alleviating neuropathic pain [39, 74]. A significant limitation of BM-MSCs, however, is that their isolation requires an invasive procedure, and their proliferation capacity and differentiation potential decline with donor age [75].

Adipose tissue represents an increasingly popular alternative source due to its abundance and ease of access through minimally invasive liposuction procedures. Adipose-derived mesenchymal stem cells (AD-MSCs) can be obtained in large quantities and have shown robust therapeutic effects. For instance, AD-MSCs have been shown to modulate microglial polarization, alleviate neuroinflammation in TBI, and reverse nociceptive hypersensitivity in experimental neuropathy [7, 76]. Stromal vascular fraction cells derived from adipose tissue, which contain AD-MSCs, have been evaluated in a pilot clinical trial for PD, demonstrating a good safety profile and potential for clinical improvement [77].

Perinatal tissues, such as the umbilical cord, placenta, and amniotic fluid, are considered valuable sources of young, highly proliferative MSCs with low immunogenicity [78]. Human umbilical cord mesenchymal stem cells (UC-MSCs) are readily available from discarded tissue, minimizing ethical concerns, and have demonstrated significant therapeutic potential. They have been shown to ameliorate neuroinflammation in depression models, protect BBB integrity in ischemic stroke, and regulate the Treg/Th17 balance in stroke patients [55, 79, 80]. MSCs from the umbilical cord’s Wharton’s Jelly have shown efficacy in AD models by reducing Aβ deposition and neuroinflammation, and are being investigated in clinical trials for TBI [81, 82].

Other sources are also being explored. Dental stem cells, particularly those from human exfoliated deciduous teeth and dental pulp, are of neuroectodermal origin and may possess superior neuroprotective capabilities compared to mesoderm-derived MSCs [83]. Exosomes from human exfoliated deciduous teeth have been shown to shift microglia M1/M2 polarization in a TBI model, while dental pulp MSC-derived exosomes have shown promise in alleviating subarachnoid hemorrhage (SAH) by inhibiting microglial pyroptosis [37, 84]. Similarly, olfactory mucosa MSCs (OM-MSCs), also of neural crest origin, are being investigated for their therapeutic potential in AD, SAH, and SCI [85–87]. The choice of MSC source may significantly impact the therapeutic outcome, and comparative studies are essential to determine the optimal cell type for specific neurological conditions.

### Key components of the MSC secretome

**Fig. 3:**
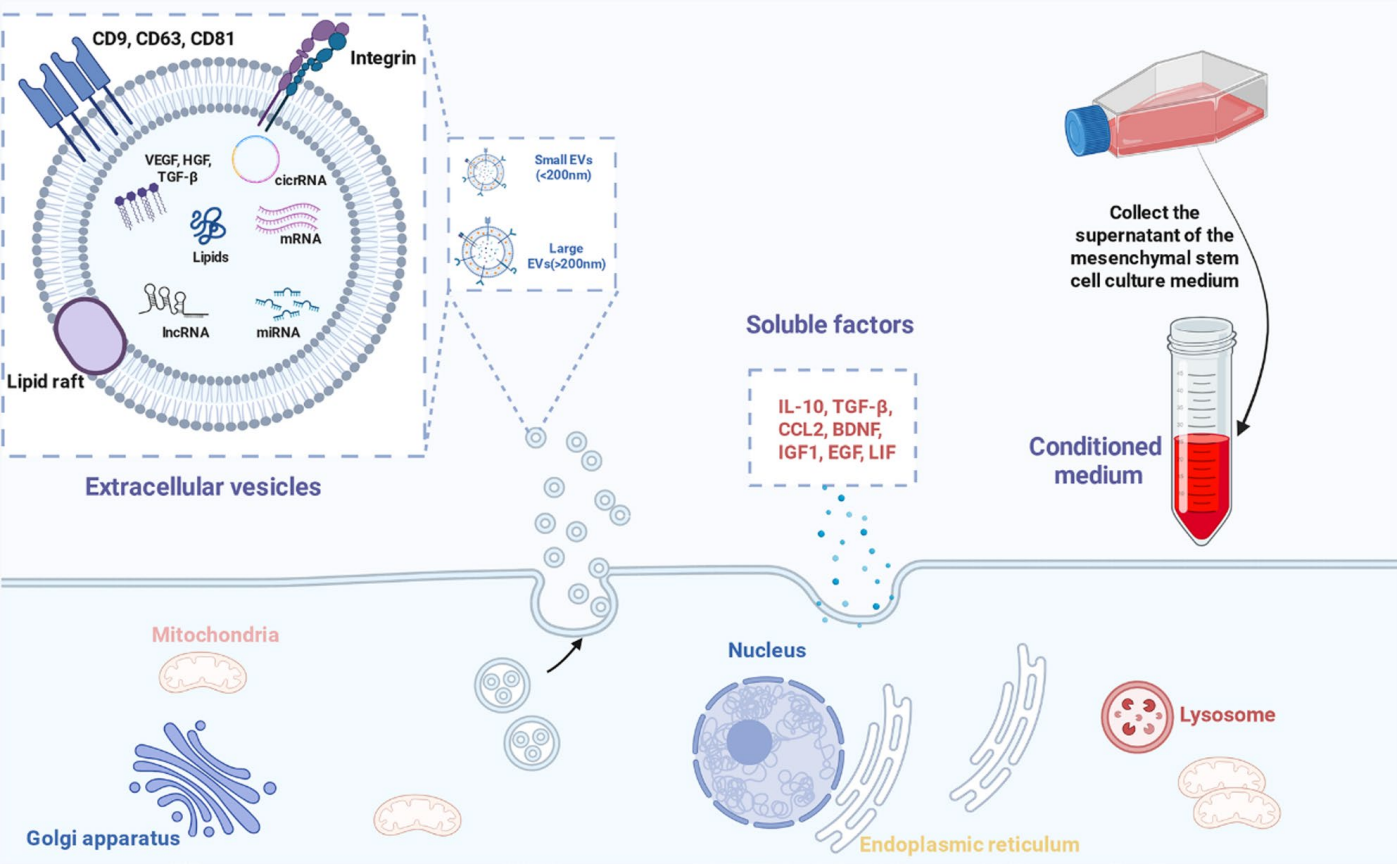
Composition and therapeutic components of the mesenchymal stem cell (MSC) secretome. The therapeutic effects of MSCs are primarily mediated by their paracrine secretome, which is actively released by the cell into the extracellular space. This secretome consists of two main bioactive fractions. (Left panel) Extracellular vesicles (EVs) are membrane-bound particles containing therapeutic cargo, including proteins, lipids, and non-coding RNAs (miRNA, lncRNA, circRNA), ranging from small EVs (< 200 nm, including exosomes) to larger EVs, that function as natural nanocarriers. (Center panel) Soluble factors are a milieu of freely secreted anti-inflammatory cytokines (e.g., IL-10, TGF-β), chemokines (e.g., CCL2), and neurotrophic factors (e.g., BDNF) that modulate the local tissue microenvironment. The entire secretome, containing the synergistic combination of both EVs and soluble factors, can be collected from the MSC culture supernatant as (right panel) conditioned medium, providing a cell-free therapeutic cocktail. CD, cluster of differentiation; VEGF, vascular endothelial growth factor; HGF, hepatocyte growth factor; TGF-β, transforming growth factor-β; circRNA, circular RNA; miRNA, microRNA; lncRNA, long non-coding RNA; EVs, extracellular vesicles; IL-10, interleukin-10; CCL2, C-C motif chemokine ligand 2; BDNF, brain-derived neurotrophic factor; IGF1, insulin-like growth factor 1; EGF, epidermal growth factor; LIF, leukemia inhibitory factor. Figure created with BioRender.com

The therapeutic effects of MSCs are largely mediated by the complex array of bioactive molecules they secrete into their microenvironment. This secretome is a dynamic mixture of paracrine factors that work in concert to modulate cellular responses in recipient tissues [88]. As conceptually detailed in Fig. 3, this secretome can be broadly divided into two critical components. The first is a diverse population of EVs, which are membrane-bound particles that serve as natural nanocarriers for a complex cargo of therapeutic proteins, lipids, and nucleic acids. The second component is a rich milieu of soluble factors, including anti-inflammatory cytokines and neurotrophic factors, that are released directly into the extracellular space. The complete secretome, often collected as conditioned medium from MSC cultures, represents the full spectrum of these secreted paracrine factors and offers a comprehensive, cell-free therapeutic approach.

### EVs: exosomes and microvesicles

EVs are lipid bilayer-enclosed particles released by cells that play a crucial role in intercellular communication by transferring a cargo of proteins, lipids, and nucleic acids to target cells [89]. They are broadly categorized based on their size and biogenesis. Microvesicles are larger vesicles (100–1000 nm) that bud directly from the plasma membrane, while exosomes are smaller vesicles (30–150 nm) originating from the endosomal pathway. Small EVs (sEVs), a term often used to encompass exosomes and smaller microvesicles (≤ 200 nm), are particularly studied for their therapeutic potential [90]. MSC-derived EVs retain many of the therapeutic properties of their parent cells, such as immunomodulatory and regenerative capabilities, and are considered key mediators of the paracrine effects of MSCs [91]. Their small size allows them to bypass biological barriers, including the BBB, to deliver their neuroprotective cargo directly to injured regions of the CNS [92]. This cargo includes a diverse array of bioactive molecules, such as neuroprotective factors, immunosuppressive proteins, and, critically, non-coding RNAs like miRNAs, which can modulate gene expression in recipient cells to reduce inflammation, inhibit apoptosis, and promote regeneration [19].

### Soluble factors: cytokines, chemokines, and growth factors

In addition to EVs, the MSC secretome contains a vast array of soluble factors that contribute significantly to its therapeutic action. These include anti-inflammatory cytokines (e.g., IL-10, TGF-β), chemokines (e.g., CCL2), and various growth and neurotrophic factors (e.g., brain-derived neurotrophic factor (BDNF), insulin-like growth factor 1, epidermal growth factor, leukemia inhibitory factor) [93, 94]. These secreted molecules exert pleiotropic effects, modulating the immune system, promoting cell survival, stimulating angiogenesis, and supporting neurogenesis. For instance, MSCs have been shown to secrete the anti-inflammatory protein tumor necrosis factor-α-stimulated gene 6 (TSG-6), which can attenuate neuropathic pain by inhibiting the TLR2/MyD88/NF-κB signaling pathway in spinal microglia [95]. Another example is the adiponectin paralog CTRP3, which BM-MSCs secrete to alleviate microglial pyroptosis after ICH [74]. Metabolomic profiling of the MSC secretome has identified key bioactive metabolites, such as certain prostaglandins and kynurenine, which have been shown to confer long-term neuroprotection in TBI models [96]. The concerted action of these soluble factors creates a microenvironment conducive to repair and regeneration, highlighting their importance within the therapeutic cocktail of the secretome.

### Conditioned medium as a therapeutic cocktail

The complete secretome, often collected as conditioned medium from MSC cultures, represents the full spectrum of secreted paracrine factors and offers a comprehensive therapeutic approach. Administering conditioned medium allows for the synergistic action of both EVs and soluble factors. Studies have shown that MSC-CM can stimulate endogenous repair, promote angiogenesis, and reduce apoptosis and neuroinflammation [97]. For example, conditioned medium from human UC-MSCs was shown to inhibit microglial activation and ameliorate neuroinflammation in ALS models, while conditioned medium from amniotic cells protected against striatal degeneration in a model of HD by reducing microglial activation [41, 98]. Even a low-molecular-weight fraction (< 700 Da) of the secretome, containing key metabolites like prostaglandins and kynurenine, has demonstrated neuroprotective effects comparable to the total conditioned medium, providing long-term functional benefits in TBI models [96]. The use of conditioned medium represents a straightforward method to harness the complete therapeutic potential of the MSC secretome for treating neurological disorders.

### Rationale for cell-free therapy

The shift toward cell-free therapies based on the MSC secretome is driven by several significant advantages over traditional whole-cell transplantation. First and foremost is an improved safety profile. Cell-free preparations, such as isolated EVs or conditioned medium, eliminate the risks associated with administering live cells, including immune rejection, unwanted differentiation, and potential tumorigenicity. This safety advantage is particularly relevant for allogeneic therapies, where the low immunogenicity of EVs offers a distinct advantage [99]. Second, cell-free products present fewer logistical and manufacturing challenges. They are easier to produce, standardize, store, and transport compared to live cells, which require complex culture and preservation protocols. The ability to characterize and quantify the bioactive components allows for better dose control and quality assurance [100]. Third, the small size of EVs, particularly exosomes, allows them to efficiently cross biological barriers like the BBB, which is a major obstacle for cell-based therapies. This small size enables their therapeutic payload to reach the CNS even with systemic administration, a major challenge for whole-cell therapies that struggle to cross the BBB [101]. Evidence from preclinical models confirms that systemically administered EVs can reach injured parts of the CNS and exert their therapeutic effects [91, 102]. These advantages position the MSC secretome as a promising, safer, and more practical alternative for clinical translation in the treatment of neurological disorders.

## MSC and secretome-based therapies for modulating neuroinflammation

MSCs have emerged as a powerful therapeutic tool for neurological disorders, largely due to their profound ability to modulate the complex neuroinflammatory environment that drives disease progression [6]. Rather than functioning primarily through cell replacement, MSCs exert their beneficial effects by orchestrating a sophisticated, multimodal response that rebalances the immune system, protects vulnerable neural tissue, and fosters a pro-regenerative milieu [103]. Their therapeutic action is dynamic and context-dependent; MSCs can sense inflammatory cues from the injured CNS and, in response, deploy a range of anti-inflammatory and immunomodulatory strategies. This includes the secretion of soluble paracrine factors, direct modulation of glial and immune cell phenotypes, and regulation of both central and peripheral immune responses [55, 104, 105]. Through these integrated mechanisms, MSCs can effectively interrupt the vicious cycle of chronic neuroinflammation and shift the local environment from one of neurotoxicity and degeneration to one of resolution and repair.

### Paracrine signaling by MSCs

#### Bioactive protein cargo

The cornerstone of MSC-mediated immunomodulation is their robust paracrine activity—the secretion of a diverse array of bioactive molecules that act on surrounding cells to quell inflammation and promote tissue homeostasis. This secretome contains a powerful cocktail of anti-inflammatory proteins, cytokines, and other small molecules that collectively orchestrate a therapeutic response [3].

One of the most critical secreted factors is TSG-6, a potent anti-inflammatory protein that is upregulated in MSCs upon licensing by inflammatory stimuli like TNF-α [106]. Studies have shown that TSG-6 is a pivotal mediator of MSCs’ therapeutic effects in various models of CNS injury. For example, in experimental TBI, MSCs inhibit microglial pyroptosis, an inflammatory form of cell death, via the secretion of TSG-6, which acts on the NLRP3/Caspase-1/GSDMD signaling pathway [107]. Similarly, in models of neuropathic pain, intrathecally administered BM-MSCs alleviate pain and neuroinflammation by secreting TSG-6, which in turn inhibits the TLR2/MyD88/NF-κB pathway in spinal microglia [95]. The importance of TSG-6 is underscored by findings that silencing its expression in MSCs significantly diminishes their therapeutic efficacy.

In addition to TSG-6, MSCs secrete canonical anti-inflammatory cytokines, most notably IL-10 and TGF-β [108]. IL-10 is a powerful immunosuppressive cytokine that can inhibit the production of pro-inflammatory cytokines by activated microglia and macrophages, thereby dampening the inflammatory cascade [81, 109]. Genetically engineering MSCs to overexpress IL-10 has been shown to enhance their therapeutic potential, leading to a greater reduction in pro-inflammatory markers and a more pronounced shift toward an anti-inflammatory state in models of TBI [110]. Likewise, TGF-β plays a crucial role in promoting the differentiation of Tregs and suppressing effector T cell responses [56]. It has also been identified as a key factor in MSC-mediated polarization of microglia to a protective phenotype. For instance, hypoxia-preconditioned MSCs were found to improve functional recovery in PD models, a process mediated by the secretion of TGF-β1, which regulated microglia immune function and autophagy [111, 112].

MSCs also produce other important immunomodulatory molecules, including prostaglandins, particularly prostaglandin E2 (PGE2). PGE2 can act on various immune cells to suppress inflammatory responses; for instance, it can inhibit T-cell proliferation and promote the M2 polarization of macrophages [113]. The level of PGE2 secretion by MSCs has even been proposed as a potency marker to predict their therapeutic efficacy in TBI, correlating directly with their ability to modulate the immune response [114]. Other secreted factors include kynurenine, identified alongside prostaglandins as a key bioactive metabolite in the MSC secretome mediating neuroprotection in TBI, and neurotrophic factors like glial cell-derived neurotrophic factor, which can directly support neuronal survival while also contributing to the modulation of microglial polarization [39, 96]. Collectively, this rich secretome allows MSCs to launch a multipronged attack on neuroinflammation, simultaneously inhibiting pro-inflammatory pathways and promoting mechanisms of resolution and repair.

### Non-coding RNAs

miRNAs are small non-coding RNAs that regulate gene expression post-transcriptionally, typically by binding to the 3’ untranslated region of target mRNAs, leading to their degradation or translational repression. A single miRNA can target hundreds of mRNAs, allowing them to act as master regulators of entire signaling networks. MSC exosomes are highly enriched in specific miRNAs that have potent anti-inflammatory and neuroprotective functions [115].

Numerous studies have identified specific exosomal miRNAs and their roles. For example, in TBI, MSC exosomes deliver miR-26a-5p, which suppresses NETs formation and modulates microglial polarization via the TAB2 (TGF-β activated kinase 1 binding protein 2)/JNK/activator protein 1 (AP1) pathway [116]. In an ICH model, human AD-MSC exosomes deliver miR-342-3p, which targets formyl peptide receptor 1 to alleviate neuroinflammation [117]. In ischemic stroke, exosomal miR-146a-5p from human UC-MSCs targets the IRAK1/TRAF6 signaling pathway to reduce microglial inflammation [118]. In SCI, exosomal miR-21a-5p targets pellino E3 ubiquitin protein ligase 1 to promote autophagy and inhibit pyroptosis, while miR-24-3p targets mitogen-activated protein kinase 9 (MAPK9) to inhibit the JNK pathway [119, 120]. In a model of sporadic AD, miR-223-3p carried by MSC sEVs directly targets and suppresses NLRP3 [121]. In neuropathic pain models, exosomal miR-26a-5p targets Wnt5a, and miR-99b-3p promotes microglial autophagy [122]. This growing list of therapeutic miRNAs highlights their central role in the paracrine action of MSCs, acting as precise molecular messengers to reprogram the inflammatory response in recipient cells.

Beyond miRNAs, other classes of non-coding RNAs within MSC exosomes are also emerging as important regulators of neuroinflammation. Long non-coding RNAs (lncRNAs) and circular RNAs can act as molecular sponges for miRNAs or interact with proteins to modulate gene expression. BM-MSC-exosomal lncRNA H19 was shown to modulate LPS-stimulated microglial M1/M2 polarization by sponging miR-29b-3p, thereby alleviating inflammation-mediated neurotoxicity [123]. In a model of cerebral ischemia/reperfusion injury, BM-MSC exosomes delivered the lncRNA KLF3-AS1, which stabilized the Sirtuin 1 protein by sponging miR-206 to upregulate the deubiquitinase USP22, ultimately reducing inflammatory injury [124].

Circular RNAs are also functionally active cargo. Exosomes from adipose-derived stem cells were found to ameliorate TBI-induced nerve damage by delivering circ-Scmh1, which promotes microglial M2 polarization by sponging miR-154-5p and regulating signal transducer and activator of transcription 6 (STAT6) [125]. In SCI, BM-MSC exosomes attenuated inflammasome-related pyroptosis by delivering circ_003564, which was shown to be critical for the exosome’s therapeutic effect [126]. The discovery of these additional RNA species expands the repertoire of mechanisms through which MSC exosomes exert their therapeutic effects and offers new targets for engineering more potent cell-free therapies.

### Modulation of glial cell phenotypes

A central mechanism through which MSCs and their secreted factors combat neuroinflammation is their ability to reprogram the functional state of resident glial cells, steering them away from neurotoxic phenotypes and toward neuroprotective and pro-regenerative ones [127, 128]. This phenotypic modulation of both microglia and astrocytes is critical for transforming the hostile post-injury microenvironment into one that supports neuronal survival and repair [129].

The most consistently reported effect of MSC therapy is the modulation of microglial polarization, specifically promoting a shift from the pro-inflammatory M1 phenotype to the anti-inflammatory M2 phenotype [130]. In numerous preclinical models of neurological disorders—including SCI, TBI, stroke, and AD—transplantation of MSCs or administration of their derivatives leads to a marked decrease in M1 markers (e.g., iNOS, CD86, TNF-α, IL-1β) and a concurrent increase in M2 markers (e.g., Arginase-1, CD206, IL-10) in the affected CNS tissue [37, 131, 132]. For example, in a rat model of ICH, human AD-MSC exosomes were shown to promote the M1-to-M2 transition, which improved neurological function and reduced neuronal apoptosis. This shift is not just a passive consequence of reduced inflammation but an active process driven by MSC-derived factors [117]. In addition, MSCs can secrete glial cell-derived neurotrophic factor, which promotes the M2 phenotype by activating the PI3K/Akt signaling pathway while inhibiting the pro-inflammatory NF-κB pathway in microglia [39]. The conditioned medium from UC-MSCs was also found to modulate the microglial secretome, thereby preventing the subsequent conversion of astrocytes into a neurotoxic state in an ALS model [41]. This modulation of microglia has profound functional consequences, as it not only reduces the production of neurotoxic molecules but also enhances the clearance of pathological debris, such as Aβ plaques in AD models [42]. The development of novel nanosystems, such as MSC membrane-camouflaged nanoparticles, further capitalizes on this mechanism, showing efficacy in PD models by promoting the transition of microglia to an anti-inflammatory phenotype [133]. While many studies utilize the M1/M2 framework, the therapeutic potential of MSCs likely extends to fine-tuning more complex, disease-specific signatures. For instance, a key area for future investigation will be to understand how MSCs interact with the dual-natured role of disease-associated microglia—specifically, how they might influence the balance between their neurotoxic inflammatory activities and their beneficial phagocytic functions necessary for clearing pathological protein aggregates [134, 135]. While direct experimental evidence for this is still emerging, this hypothesis provides a new lens through which to evaluate the mechanisms of MSC-based therapies.

In parallel with their effects on microglia, MSCs also regulate the reactivity of astrocytes. Following CNS injury, astrocytes can adopt a detrimental, neurotoxic A1 phenotype or a beneficial, neuroprotective A2 phenotype. MSC-based therapies have been shown to suppress the formation of A1 astrocytes and promote their polarization toward the A2 state. In a mouse model of neuroinflammation, MSC transplantation reversed the expression of extracellular matrix components and altered immune cell infiltration, indicating a broad effect on the CNS environment that includes astrocyte modulation [136]. A direct mechanism was elucidated in a study showing that peripheral blood-derived MSCs induce the polarization of astrocytes to the A2 phenotype through the secretion of TGF-β, which activates the PI3K/Akt signaling pathway in astrocytes [137]. Similarly, hypoxic-preconditioned MSC-derived EVs promote the conversion of A1 to A2 astrocytes in SCI models, an effect mediated by the transfer of miR-21, which targets the JAK2/STAT3 pathway [138]. By preventing A1 astrocyte activation, MSCs reduce a significant source of neurotoxicity and restore the supportive functions of astrocytes [128]. This dual control over both microglial and astrocytic phenotypes is a powerful therapeutic action, enabling MSCs to comprehensively re-engineer the glial response from one that perpetuates damage to one that actively facilitates healing and neuroprotection.

### Regulation of peripheral immune cell responses

The disruption of the BBB is a critical event in many CNS pathologies that allows for the influx of peripheral immune cells and inflammatory molecules, thereby exacerbating neuroinflammation. MSC-based therapies restore and preserve BBB integrity through multiple mechanisms targeting the cellular and molecular components of the neurovascular unit, a crucial function that physically limits the entry of peripheral inflammatory cells. MSCs have been shown to reduce BBB permeability and decrease brain edema in models of stroke, TBI, and subarachnoid hemorrhage [48, 139]. They accomplish this by suppressing the activity of MMPs, particularly MMP-9, which are enzymes that degrade tight junction proteins and the basal lamina of the BBB [140]. In diabetic mouse models with associated emotional deficits, MSC administration suppressed MMP-9 activity, leading to the upregulation of tight junction proteins claudin-5 and occludin and the restoration of BBB integrity [140].

Furthermore, MSCs can directly influence the expression of factors that regulate vascular permeability. In a model of experimental autoimmune encephalomyelitis (EAE), MSCs engineered to secrete the anti-aging protein Klotho were more effective than unmodified MSCs at reducing BBB permeability, an effect accompanied by decreased levels of adhesion molecules (ICAM-1, VCAM-1) and MMP-9 in the brain [49]. Similarly, in ischemic stroke, MSCs overexpressing fibroblast growth factor 21 significantly mitigated BBB disruption by inhibiting the loss of tight junction proteins and suppressing the upregulation of aquaporin 4, a water channel involved in cerebral edema [48].

Complementing their direct effects on the BBB, MSCs also systemically regulate the activity of peripheral immune cells, whose infiltration across a compromised BBB is a key driver of secondary injury and chronic neuroinflammation [141]. By systemically altering the immune response, MSCs can reduce the influx of inflammatory cells into the brain and spinal cord and promote a more tolerogenic peripheral environment [142].

A primary target of MSC-mediated immunomodulation is the T lymphocyte population. MSCs are known to exert a powerful influence on T-cell polarization, tipping the balance away from pro-inflammatory lineages and toward anti-inflammatory or regulatory ones [143]. Specifically, MSCs can suppress the differentiation and function of pro-inflammatory Th17 cells, which are key drivers of pathology in autoimmune diseases like MS and also contribute to damage in acute CNS injuries [144, 145]. Concurrently, MSCs promote the proliferation and function of Tregs, an immunosuppressive T-cell subset crucial for maintaining immune tolerance and resolving inflammation [146]. In a model of acute ischemic stroke, UC-MSCs were shown to mitigate the pathological shift in T-cell compartments, correcting the Th17/Treg imbalance observed after stroke. This effect was dependent on mitochondrial transfer from MSCs to T-cells, which restored mitochondrial function and reversed the pro-inflammatory T-cell phenotype [55]. Similarly, in TBI, UC-MSCs were found to facilitate the trans-differentiation of Th17 cells into Tregs, a process mediated through the TGF-β/Smad3/NF-κB signaling pathway, which correlated with improved neurological recovery [56]. By promoting a systemic shift toward a Treg-dominant environment, MSCs help to create a state of peripheral tolerance that limits autoimmune-mediated attacks on the CNS.

MSCs and their derivatives also exert direct effects on cells of the innate immune system, such as neutrophils. In acute CNS injuries like TBI and stroke, neutrophils are among the earliest and most aggressive infiltrators, contributing significantly to secondary damage through the release of cytotoxic granules and the formation of NETs. Recent studies have revealed that MSC-derived therapies can effectively curb this detrimental neutrophil activity. For instance, MSC-derived exosomes have been shown to mitigate TBI by inhibiting the formation of NETs [116]. The supernatant from human MSCs, when delivered intranasally after ischemic stroke, was found to reduce neutrophil infiltration into the brain and promote the polarization of neutrophils toward an anti-inflammatory “N2” phenotype, which is associated with increased production of IL-10 and TGF-β. This polarization was driven by the activation of the PPAR-γ/STAT6/SOCS1 pathway in neutrophils [147]. By regulating both the adaptive and innate arms of the peripheral immune system, MSCs provide a comprehensive immunomodulatory therapy that addresses inflammation both within and outside the CNS, thereby preventing the amplification of the initial injury and fostering an environment conducive to long-term recovery.

### Targeting key intracellular signaling pathways

The ability of MSCs to modulate glial phenotypes and exert broad anti-inflammatory effects is rooted in their capacity to target fundamental intracellular signaling pathways that govern the immune response [148]. The MSC secretome contains a multitude of factors that can simultaneously inhibit pro-inflammatory cascades while activating pro-survival and anti-inflammatory pathways in target cells [149–151].

### Suppression of TLR signaling

TLRs are pattern recognition receptors that play a crucial role in initiating the innate immune response by recognizing pathogen-associated molecular patterns and DAMPs. TLR signaling, particularly through TLR4, is a primary trigger for NF-κB and MAPK activation and subsequent neuroinflammation. The secretome of MSCs can effectively interfere with this upstream signaling. In a model of ICH, human UC-MSC exosomes were shown to inhibit inflammation and fibrotic scar formation by downregulating the pentraxin 3 (PTX3)/TLR4/NF-κB pathway [152]. Menstrual blood-derived endometrial stem cells were also found to suppress neuroinflammation by regulating microglia through the TLR4/MyD88/NLRP3/Casp1 pathway [153]. In neuropathic pain, the analgesic effect of BM-MSC-secreted TSG-6 was attributed to the inhibition of the TLR2/MyD88/NF-κB pathway in spinal microglia [95]. By targeting these key sentinel receptors, MSCs can prevent the very initiation of the inflammatory cascade in response to injury-related signals.

### Inhibition of pro-inflammatory pathways: NF-κB, MAPK, and JNK

The NF-κB pathway is a master regulator of inflammation, controlling the transcription of numerous pro-inflammatory cytokines, chemokines, and enzymes like iNOS and cyclooxygenase-2. Its activation is a key feature of neuroinflammation across a wide range of CNS disorders [154]. MSCs and their derivatives are potent inhibitors of NF-κB signaling. In ICH models, human UC-MSC exosomes inhibited the TLR4/NF-κB pathway, thereby reducing the expression of TNF-α and IL-1β [152, 155]. Similarly, in SCI, MSC-derived exosomal miR-21a-5p was found to inhibit pyroptosis by suppressing the NF-κB pathway, while in depression models, docosapentaenoic acid-enhanced exosomes targeted the MyD88/TRAF6/NF-κB pathway [119, 156]. The MAPK family, including p38 MAPK and JNK, are also critical signaling nodes that translate extracellular stresses into inflammatory and apoptotic responses [157]. MSC-based therapies have been shown to effectively suppress these pathways. For instance, human UC-MSC exosomes mitigate post-stroke neuroinflammation by delivering high mobility group box 1 (HMGB1), which in turn inhibits a triggering receptor expressed on myeloid cells 1 (TREM1)-dependent NF-κB/p38 MAPK activation axis in microglia [35]. In TBI, intranasally administered MSC EVs prevent chronic brain dysfunction by inhibiting NLRP3-p38/MAPK signaling [158]. Furthermore, exosomal miRNAs from MSCs, such as miR-24-3p, can inhibit the JNK pathway by targeting MAPK9, thereby reducing apoptosis and inflammation in SCI [120]. By concurrently targeting these central pro-inflammatory pathways, MSCs can effectively shut down the production of a wide array of detrimental mediators.

### Attenuation of inflammasome activation: The NLRP3 axis

The inflammasome is a multi-protein complex that, upon sensing cellular danger signals, activates caspase-1, leading to the processing and release of the highly pro-inflammatory cytokines IL-1β and IL-18, and initiating a form of inflammatory cell death called pyroptosis. The NLRP3 inflammasome, in particular, has been implicated as a key driver of neuroinflammation in AD, TBI, SCI, and stroke [67]. MSCs and their exosomes are powerful regulators of this pathway. In a model of sporadic AD, MSC sEVs were shown to alleviate NLRP3/GSDMD-mediated neuroinflammation [121]. In SCI, tanshinone IIA-pretreated UC-MSC exosomes suppressed NLRP3 inflammasome activation by delivering miR-223-5p, which inhibits the deubiquitinase USP8, leading to NLRP3 degradation [131]. Similarly, in TBI, AD-MSC exosomes were found to ameliorate inflammatory activation by reducing NLRP3 inflammasome secretion by microglia [159]. In diabetic ICH, BM-MSC EVs carrying miR-183-5p targeted programmed cell death 4 (PDCD4) to repress the NLRP3 pathway [160]. This direct inhibition of the inflammasome provides a potent mechanism to block the maturation of key inflammatory cytokines and prevent pyroptotic cell death, thereby limiting the amplification of the inflammatory cascade.

### Pleiotropic effects beyond direct immunomodulation

While the direct modulation of glial cells and inflammatory pathways is a cornerstone of MSC therapy, the therapy’s beneficial impact extends to a wide range of pleiotropic effects that collectively contribute to neuroprotection and tissue repair.

### Alleviating oxidative stress and mitochondrial dysfunction

Oxidative stress, resulting from an imbalance between the production of ROS and the capacity of antioxidant defenses, is one of the major contributors to neuronal damage in both acute and chronic neurological disorders [161]. Mitochondrial dysfunction is often both a cause and a consequence of this oxidative stress [162]. MSC-based therapies can effectively counteract these processes. The MSC secretome has demonstrated the ability to reduce oxidative stress, improve mitochondrial function, and decrease apoptosis in models of diabetic cognitive impairment [163]. MSC-derived small EVs have been shown to exert antioxidant effects by activating the nuclear factor erythroid 2-related factor 2 pathway, a master regulator of antioxidant responses [164]. Bioengineering approaches, such as coating ROS-scavenging nanoparticles with MSC membranes, can leverage both the targeting ability of the MSC and the antioxidant properties of the core particle to alleviate oxidative stress and modulate mitochondrial dysfunction in models of PD [133]. Furthermore, MSCs can directly transfer healthy mitochondria to damaged cells, a mechanism that helps restore mitochondrial function and reverse shifts in T-cell compartments in models of ischemic stroke [55].

### Promoting endogenous repair

Beyond protecting existing cells, MSCs and their secretome actively promote the brain’s innate capacity for self-repair. They have been shown to stimulate neurogenesis, angiogenesis, and synaptic plasticity [165]. In stroke models, MSC therapy has been linked to increased endogenous neurogenesis and angiogenesis, contributing to long-term functional recovery [80, 166]. Exosome treatment after TBI significantly enhances both angiogenesis and neurogenesis in the lesion boundary zone and the dentate gyrus, a key neurogenic niche [167, 168]. One of the causes for this pro-regenerative effect is the secretion of various neurotrophic and growth factors, such as BDNF and vascular endothelial growth factor. By fostering this triad of repair processes, MSC therapies help to rebuild damaged neural circuits.

### Facilitating clearance of pathological protein aggregates

In many neurodegenerative diseases, the accumulation of misfolded protein aggregates—such as Aβ and tau in AD, and α-synuclein in PD—is a central pathological feature that drives neurotoxicity and neuroinflammation [169]. MSC-based therapies have shown promise in facilitating the clearance of these toxic proteins. In AD mouse models, MSC transplantation has been shown to reduce Aβ deposition. This clearance can be achieved through multiple mechanisms, including the enhancement of microglial phagocytic activity, the secretion of Aβ-degrading enzymes, and the promotion of autophagy [170–172]. For example, the administration of human adult ischemia-tolerant MSCs was shown to increase the expression of several Aβ-degrading enzymes in the brain [173]. Similarly, in a PD model, the secretome of neural-induced AD-MSCs was able to decrease the aggregation of α-synuclein and improve the clearance of these aggregates through the restoration of autophagic flux [174]. By aiding the removal of these primary pathological drivers, MSCs can help to break the cycle of neurotoxicity and inflammation.

## Therapeutic applications of MSCs and their secretome across neurological disorders

**Fig. 4:**
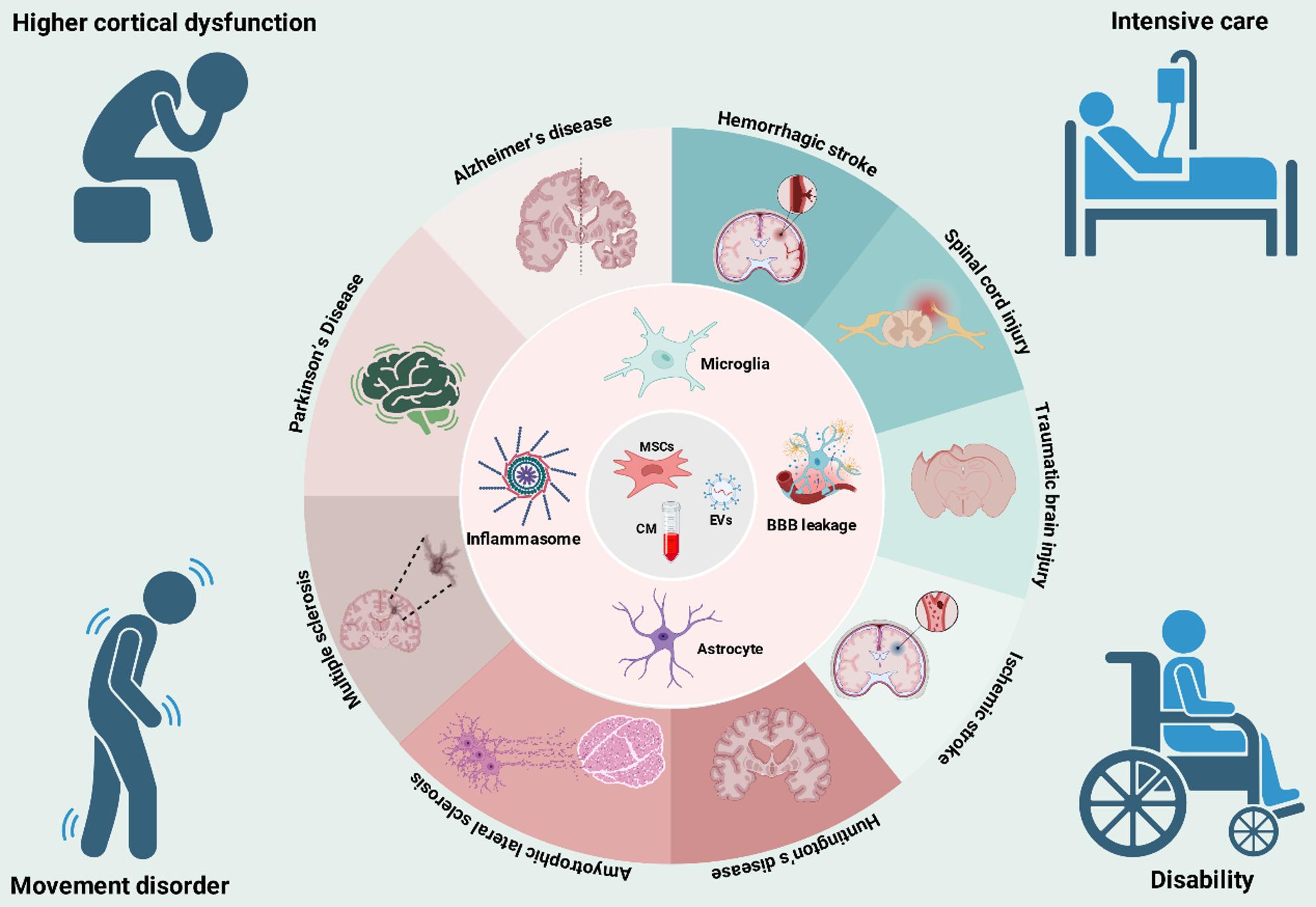
Therapeutic applications of MSCs and their secretome across a spectrum of neurological disorders. MSCs and their secretome represent a therapeutic strategy for a broad range of neurological disorders. They act by targeting core pathologies, including glial cell activation (microglia, astrocytes), inflammasome activity, and blood-brain barrier leakage. This approach is applicable to chronic neurodegenerative diseases (e.g., Alzheimer’s, Parkinson’s) and acute CNS injuries (e.g., stroke, spinal cord injury, traumatic brain injury). The therapeutic goal is to mitigate severe clinical outcomes, including higher cortical dysfunction, movement disorders, and long-term disability. BBB, blood-brain barrier; CM, conditioned medium; EVs, extracellular vesicles; MSCs, mesenchymal stem cells. Figure created with BioRender.com

The potent neuroprotective and immunomodulatory properties of MSCs and their secretome have been extensively evaluated in a wide range of preclinical models of neurological disease. The therapeutic applications span chronic neurodegenerative diseases, where the goal is to slow relentless progression, and acute CNS injuries, where the focus is on mitigating secondary damage and promoting repair (Fig. 4).

### Chronic neurodegenerative diseases

Chronic neurodegenerative diseases are characterized by the progressive loss of specific neuronal populations, leading to debilitating cognitive, motor, or sensory decline. Neuroinflammation is a common feature that accelerates this neurodegenerative process. MSC-based therapies offer a promising approach by targeting this inflammatory component while simultaneously providing neurotrophic support.

### Alzheimer’s disease

AD is defined by the extracellular deposition of Aβ plaques and intracellular formation of neurofibrillary tangles (NFTs) from hyperphosphorylated tau protein, which together drive neuroinflammation and neuronal death [175]. MSC therapies have shown efficacy in targeting multiple facets of AD pathology. Transplantation of human amniotic MSCs into amyloid precursor protein / presenilin 1 (APP/PS1) transgenic mice was found to reduce Aβ deposition, rescue spatial learning and memory deficits, and enhance hippocampal neurogenesis and synaptic plasticity [170]. Similarly, Wharton’s Jelly-derived MSCs improved memory and reduced Aβ levels in APP/PS1 mice, an effect attributed to the modulation of neuroinflammation, including a shift from pro-inflammatory to anti-inflammatory microglial activation [81]. The combination of unmodified MSCs and hematopoietic stem cells reduced amyloid plaque density and astrogliosis, improved behavioral deficits, and suppressed IL-6 production in human monocytes, highlighting anti-inflammatory mechanisms [176].

The MSC secretome provides a cell-free approach for the treatment of AD. Research has shown that exosomes from BM-MSCs can improve AD-like behaviors and reduce hippocampal expression of Aβ and phosphorylated tau, an effect associated with the regulation of glial activation and upregulation of BDNF [177]. In a streptozotocin-induced AD model, induced pluripotent stem cell (iPSC)-MSC-derived sEVs alleviated NLRP3/GSDMD-mediated neuroinflammation, decreased amyloid deposition, and mitigated cognitive dysfunction, with effects partially attributed to exosomal miR-223-3p [121]. Enhancing the cargo of MSCs or their exosomes can further boost efficacy. Curcumin-primed MSC exosomes showed a more significant effect on polarizing microglia to the anti-inflammatory M2 phenotype and improving learning in an AD rat model compared to naïve exosomes [178]. Furthermore, engineered exosomes co-delivering beta-secretase 1 small interfering RNA and the anti-inflammatory agent berberine effectively reduced Aβ deposition and neuroinflammation after intranasal administration [179].

### Parkinson’s disease

PD is characterized by the progressive loss of dopaminergic neurons in the substantia nigra, leading to motor deficits. Oxidative stress and neuroinflammation are key drivers of this neuronal degeneration [180]. MSC-based therapies have shown promise in protecting these vulnerable neurons. Systemic administration of human AD-MSCs was found to inhibit the differentiation of pro-inflammatory Th17 cells and promote a regulatory T cell phenotype in peripheral blood mononuclear cells from PD patients, suggesting a peripheral immunomodulatory mechanism [144]. The MSC secretome is also effective; neural-induced human AD-MSC conditioned medium and its exosomes exerted significant neuroprotection in a rotenone-induced rat model of PD by decreasing α-synuclein toxicity, inhibiting neuroinflammation and apoptosis, and restoring autophagy [174]. MSC EVs have been shown to efficiently alleviate PD-related motor deficits in animal models by suppressing inflammatory immune cell activation and enhancing the viability of dopamine-producing neurons [102]. Moreover, innovative biomimetic nanosystems, such as mesoporous polydopamine nanoparticles coated with an MSC membrane, can traverse the BBB, scavenge ROS, and promote a shift from pro- to anti-inflammatory microglial phenotypes, resulting in the protection of dopaminergic neurons [133].

### Multiple sclerosis and demyelinating disorders

MS is an autoimmune disease of the CNS characterized by chronic inflammation, demyelination, and axonal degeneration [181]. MSCs are particularly well-suited for treating MS due to their potent immunomodulatory properties. Intrathecal injection of autologous MSCs in patients with progressive MS led to a significant reduction in serum biomarkers of neurodegeneration (neurofilament light chain) and astrogliosis (glial fibrillary acidic protein), which paralleled beneficial effects on cognition and neurological function [182]. In the EAE mouse model of MS, MSCs engineered to secrete Klotho, an anti-aging protein, showed superior therapeutic efficacy compared to unmodified MSCs, more potently reducing disease severity, neuroinflammation, and BBB permeability [49]. Similarly, MSCs engineered to secrete IFN-β also demonstrated enhanced therapeutic effects in EAE models [183]. The MSC secretome is a key mediator of these effects. MSC-derived EVs have been shown to modulate astrocytes’ reactive phenotype and reduce neurotoxicity, while MSC-derived exosomes promote remyelination and reduce neuroinflammation by shifting microglial polarization to the M2 phenotype and inhibiting the TLR2/IRAK1/NF-κB pathway [184, 185]. A combination therapy using MSC-derived exosomes and an NLRP3 inflammasome inhibitor was more effective than either monotherapy in a cuprizone-induced demyelination model, enhancing myelin repair by attenuating both inflammation and oxidative stress [186].

### Amyotrophic lateral sclerosis and Huntington’s disease

ALS is a fatal neurodegenerative disease involving the progressive loss of upper and lower motor neurons. Neuroinflammation is a significant contributor to disease progression [187]. Both preclinical and clinical studies have explored MSC therapy for ALS. In the SOD1(G93A) mouse model, multiple systemic transplantations of human amniotic MSCs retarded disease progression, extended survival, improved motor function, and decreased neuroinflammation [188]. The secretome has also proven effective; UC-MSC-conditioned medium extended the lifespan of SOD1-G93A mice by inhibiting microglial activation and astrogliosis [41]. Intranasal delivery of MSC-derived sEVs in SOD1 mice also improved motor performance and survival time by inhibiting neuroinflammation and the overactivation of the complement and coagulation cascades and NF-κB signaling [189].

In HD, an autosomal dominant disorder caused by a mutation in the huntingtin gene, MSC therapy has also shown promise. In the R6/2 mouse model, intranasal administration of MSCs increased survival, attenuated behavioral disruptions, and downregulated the gene expression of inflammatory modulators [190]. Furthermore, intravenously administered MSCs were shown to restore expression of the astrocytic AQP-4 water channel, which is involved in the brain’s glymphatic clearance system. This improved the brain distribution of therapeutic antisense oligonucleotides, enhancing their ability to suppress mutant huntingtin protein [191].

### Acute CNS injuries

In acute CNS injuries, the primary mechanical or ischemic damage is followed by a devastating wave of secondary injury characterized by edema, excitotoxicity, oxidative stress, and a robust neuroinflammatory response. The primary goal of therapy is to mitigate this secondary cascade and create an environment conducive to repair and regeneration.

### Ischemic stroke

Ischemic stroke results from the obstruction of blood flow to the brain, leading to neuronal death in the ischemic core and a surrounding penumbra of salvageable tissue [192]. MSC therapy has shown multifaceted benefits in preclinical stroke models. Intravenous administration of human UC-MSCs promoted functional recovery and neuroprotection in rats, with mechanisms involving immunomodulation (increased TGF-β1 and interleukin-1 receptor antagonist), angiogenesis, and neurogenesis [80]. Transplantation of MSCs overexpressing fibroblast growth factor 21 more effectively preserved BBB integrity, reduced infarct volume, and suppressed neuroinflammation compared to unmodified MSCs [48].

Studies on MSC secretome have also demonstrated their beneficial effects on ischemic stroke. Intranasal administration of human UC-MSC exosomes led to their accumulation in ischemic brain regions, where they were internalized by microglia, improved neurological outcomes, and promoted a sustained shift toward an anti-inflammatory microglial phenotype via an HMGB1-TREM1-p38 MAPK axis [35]. BM-MSC exosomes have been shown to ameliorate cerebral ischemia/reperfusion injury by suppressing NLRP3 inflammasome-mediated pyroptosis through the modulation of microglial polarization [193]. Furthermore, human placental chorionic MSCs ameliorated cognitive dysfunction and hippocampal neuronal injury in stroke mice by reducing neuroinflammation and activating the MEK/ERK/CREB signaling pathway [194].

### Traumatic brain injury

TBI involves a primary mechanical injury followed by a complex secondary injury cascade, in which neuroinflammation plays a pivotal role [195]. Systemic administration of MSCs or their secretome has shown significant therapeutic potential. In rodent models, AD-MSCs improved neurocognitive outcomes and modulated neuroinflammation [196]. Intravenous administration of MSC exosomes improved sensorimotor and cognitive function, promoted endogenous angiogenesis and neurogenesis, and reduced neuroinflammation [167, 168, 197]. Intranasal delivery allows for the non-invasive administration of exosomes that inhibit NLRP3-p38/MAPK signaling and prevent chronic cognitive and mood impairments [158]. Advanced biomaterial scaffolds can enhance the efficacy of MSC-based therapies by improving cell survival and providing sustained release of therapeutic factors at the injury site [198, 199].

### Spinal cord injury

SCI results in devastating and often permanent loss of motor and sensory function below the level of injury. The secondary injury process, characterized by intense inflammation and the formation of an inhibitory glial scar, is a major barrier to axonal regeneration [200]. MSC-based therapies target these pathological processes to create a more permissive environment for repair. Transplantation of OM-MSCs was shown to improve motor function in SCI mice by regulating M1/M2 polarization of microglia [87]. Peripheral blood-derived MSCs also facilitate functional recovery by shifting microglia/macrophage plasticity towards the M2 phenotype [38]. Combination therapy, such as human UC-MSCs with ultrashort wave therapy, can synergistically attenuate neuroinflammation through the Nurr77 (also known as NR4A1)/NF-κB pathway [201].

Exosomes are increasingly recognized as a potent cell-free option for SCI. Exosomes derived from MSCs can mitigate SCI-related injury by suppressing apoptosis and inflammation [120]. Tanshinone IIA-pretreated UC-MSC exosomes enhanced neuroprotection and functional recovery by delivering miR-223-5p, which targets the USP8/NLRP3 axis to dampen neuroinflammation [131]. Hypoxia-preconditioned MSC EVs promote the transformation of reactive astrocytes from the detrimental A1 phenotype to the neuroprotective A2 phenotype by delivering miR-21, which targets the JAK2/STAT3 pathway [138]. Innovative delivery systems, such as porous microneedle patches, can provide sustained, localized delivery of EVs to the injury site, reducing scar tissue formation and promoting significant functional recovery [202].

### Hemorrhagic stroke

ICH and SAH are severe forms of stroke where blood vessel rupture leads to direct brain tissue damage and a potent inflammatory response. Human UC-MSC exosomes have been shown to inhibit inflammation and fibrotic scar formation after ICH by modulating the PTX3/TLR4/NF-κB/MMP3 pathway, leading to improved behavioral performance in rats [152]. In a diabetic ICH model, BM-MSC EVs delivered miR-183-5p to target the PDCD4/NLRP3 pathway, thereby alleviating neuroinflammation [160]. BM-MSCs can also alleviate microglial pyroptosis after ICH by secreting CTRP3, which acts via the PI3K/AKT and Syk signaling pathways [74]. In SAH models, OM-MSC-derived exosomes protect against neuroinflammation by activating mitophagy, while MSC EVs promote microglial M2 polarization via the AMP-activated protein kinase (AMPK)/NF-κB pathway [86, 203]. These findings highlight the potential of MSC-based therapies to address the specific inflammatory challenges posed by hemorrhagic stroke.

## Clinical translation

The transition of MSC-based therapies from promising preclinical findings to effective clinical treatments for neurological disorders is an area of intense research and development. While numerous studies have highlighted their potential, the path to clinical application is fraught with challenges related to safety, efficacy, standardization, and scalability. Despite these hurdles, significant progress has been made, with several clinical trials completed or underway, providing valuable insights into the feasibility and potential of these novel therapeutics.

### Overview of clinical trials in neurological disorders

Clinical trials investigating MSC therapies have been conducted for a range of neurological conditions, with most being in the early phases (Phase 1 and 2) focused on establishing safety and tolerability. A systematic review of 94 stem cell clinical trials for neurodegenerative diseases (AD, PD, ALS, and HD) revealed that MSCs are one of the most frequently used cell types [20].

For AD, a randomized, double-blind, placebo-controlled Phase 2a trial of laromestrocel, an allogeneic bone marrow-derived MSC product, demonstrated a favorable safety profile in patients with mild AD. The study met its primary safety endpoint, with no infusion-related reactions or amyloid-related imaging abnormalities. Furthermore, it provided encouraging signals of efficacy, including a slowing of whole brain and left hippocampal volume decline and improvements in composite clinical scores compared to placebo, warranting larger-scale trials [204]. Of 94 stem cell clinical trials reviewed for neurodegenerative diseases, nearly 70% of the 8000 + participants were enrolled in AD-related studies, reflecting the immense interest in this area [20].

In PD, a Phase 1 open-label, dose-escalation study assessed the safety of a single intravenous infusion of allogeneic BM-MSCs in patients with mild to moderate PD. The treatment was found to be safe, well-tolerated, and not immunogenic across four dose levels. Notably, the highest dose group showed potential signs of clinical benefit, with reductions in OFF-state Unified Parkinson’s Disease Rating Scale motor scores and peripheral inflammation markers at 52 weeks post-infusion [205]. Another pilot trial using autologous adipose-derived stromal vascular fraction cells injected in facial regions also reported a good safety profile and evidence of clinical improvement in a majority of subjects at 12 and 24 months [77]. A secondary analysis from an RCT using allogeneic BM-MSCs in older adults with PD unexpectedly found that repeated infusions were associated with improved kidney function, suggesting systemic benefits beyond the CNS [206].

MS, as a chronic autoimmune and neurodegenerative disease, is one of the prime candidates for the immunomodulatory and neuroprotective effects of MSCs. A phase II RCT in progressive MS patients treated with intrathecal autologous MSC-neural progenitors found that while the primary endpoint (Expanded Disability Status Scale Plus) was not met for the overall group, a subgroup of more disabled patients (Expanded Disability Status Scale 6.0-6.5) showed significant improvements in walking speed and bladder function, as well as a reduced rate of grey matter atrophy [207]. Another study on progressive MS patients treated with repeated intrathecal injections of autologous MSCs (MSC-NG01) found a gradual and consistent reduction in serum biomarkers of neurodegeneration (neurofilament light chain and glial fibrillary acidic protein), paralleled by improvements in cognition and functional tests [182]. A phase I/II dose-finding study in MS patients using two different intrathecal doses of UC-MSCs found both protocols to be safe and associated with improvements in general disability scales [208]. However, the immunomodulatory function of MSCs may be context-dependent; one study found that MSCs from MS patients could have a pro-inflammatory effect, increasing Th17 cells, an effect that could be modulated by blocking ADAM28 or adding IL-10 [209].

For ALS, a randomized, double-blind, placebo-controlled Phase 3 study evaluated intrathecal treatments of MSCs engineered to secrete high levels of neurotrophic factors. While the trial did not meet its primary efficacy endpoint in the overall population, a pre-specified analysis of a subgroup of patients with less severe disease at baseline suggested a potential functional benefit compared to placebo. Importantly, the treatment was well-tolerated and resulted in significant improvements in cerebrospinal fluid (CSF) biomarkers related to neuroinflammation and neurodegeneration [210]. A small pilot safety study of ten ALS patients treated with two intravenous infusions of human BM-MSCs EVs found the treatment to be safe, and 30% of subjects’ ALS functional rating scale-revised scores did not decline over a 3-month period, warranting a larger controlled study [211]. These early findings, while encouraging, are preliminary, and larger, more robust trials are needed to confirm any survival benefit. The FDA has recently updated quality measures for ALS care, which will help standardize outcome assessments in future trials [212].

For HD, an autosomal dominant neurodegenerative disorder with no approved disease-modifying therapies, MSCs are also being explored for their neuroprotective and anti-inflammatory properties. A Phase 2 randomized, double-blind, placebo-controlled trial evaluated NestaCell^®^, an allogeneic therapy based on human dental pulp stem cells, in HD patients. The study met its primary safety endpoint, demonstrating that repeated intravenous infusions of human dental pulp stem cells were well-tolerated with no increased incidence of adverse events compared to placebo. Importantly, the trial also showed statistically significant improvements in the primary efficacy endpoint, the Unified Huntington’s Disease Rating Scale Total Motor Score, for both dose groups. The higher dose group (2 million cells/kg) also demonstrated significant benefits in Total Functional Capacity. While MRI analysis showed only a non-significant trend towards slowed white and grey matter decline, the robust improvements in motor and functional outcomes support the advancement of this cell therapy to a larger Phase 3 trial to confirm its efficacy [213].

Stroke is another major target for MSC therapy. A meta-analysis of 15 randomized controlled trials (RCTs) and 15 non-randomized trials (1217 patients) found that MSC transplantation significantly improved neurological deficits (measured by National Institutes of Health Stroke Scale (NIHSS)) and activities of daily living (measured by modified Rankin Scale), and was associated with lower mortality rates [214]. Another meta-analysis of 9 RCTs (316 patients) also confirmed that MSCs significantly reduced NIHSS scores, although no significant effect was seen on the Barthel Index or modified Rankin Score in this analysis [215]. A network meta-analysis of 19 studies (1055 patients) comparing five different stem cell types found that UC-MSCs had the best efficacy in repairing neurological function (NIHSS scores), while bone marrow mononuclear cells were best for improving motor function and daily living ability [216]. The STEMTRA trial, a phase 2 study, showed that intracortical implantation of allogeneic modified BM-MSCs in patients with chronic motor deficits after ischemic stroke or TBI resulted in significant and sustained improvements in motor status [217]. For acute ICH, a phase I dose-escalation trial demonstrated that intravenous administration of allogeneic BM-MSCs was feasible and safe, paving the way for larger efficacy studies [218].

In TBI, the phase 2 STEMTRA trial, which included patients with chronic motor deficits from TBI, demonstrated that intracranial implantation of SB623 cells (modified allogeneic BM-MSCs) was safe and led to significant improvement in motor status at 24 weeks, which was sustained at 48 weeks. These findings provide Class I evidence for the efficacy of this cell therapy in chronic TBI [217]. A single case report detailed the treatment of a severe TBI patient with intravenous human BM-MSC EVs, reporting substantial and sustained improvements in functional independence with no adverse events [219]. The TRAUMACELL trial, a phase III study, is currently planned to investigate repeated intravenous treatment with Wharton’s Jelly-derived MSCs in severe TBI patients, using positron emission tomography-magnetic resonance imaging to evaluate neuroinflammation as the primary outcome, which will provide crucial data on the immunomodulatory effects of MSCs in humans [82].

SCI represents one of the most extensively studied indications for MSC therapy, with trials targeting all phases of injury. In a Phase I trial for traumatic SCI, intrathecal delivery of autologous, culture-expanded AD-MSCs was found to be safe and well-tolerated, with seven of ten patients showing improvement in American Spinal Injury Association Impairment Scale (AIS) grade at final follow-up [220]. A 9-year case series of 106 patients with complete SCI treated with combined autologous BM-MSCs and Schwann cells reported significant and consistent improvements in AIS scores (motor, light touch, and pinprick), as well as in functional independence and quality of life, regardless of injury level or duration (subacute vs. chronic) [221]. Another Phase I/II trial in chronic SCI patients using expanded autologous BM-MSCs and allogeneic UC-MSCs found both protocols to be safe, with both groups showing significant improvements in total AIS scores [222].

To facilitate a comparative overview of the clinical landscape, we have consolidated key trial characteristics and outcomes in Table 1. Collectively, these data illustrate that while the safety profile of MSCs and secretome is consistently favorable across diverse etiologies and administration routes, efficacy outcomes remain variable. This heterogeneity reflects the broad diversity in trial designs, including substantial differences in cell sources, dosages, and patient populations. These disparities underscore the critical need for standardization to improve clinical translation, the specific challenges of which—including donor variability and manufacturing consistency—are critically analyzed in the subsequent section, “Hurdles to clinical application”.

**Table 1:** Summary of key clinical trials of MSCs and secretome in neurological disorders

Disease	Phase/study type	Cell/secretome sources	Route of admin	Key findings and outcomes	NCT number	Ref(s)
AD	Phase 2a (randomized, double-blind, placebo-controlled)	Allogeneic BM-MSCs	Intravenous	Safety confirmed. Met primary safety endpoints. Efficacy signals included slowed hippocampal volume decline and improved composite clinical scores compared to placebo.	NCT05233774	[204]
PD	Phase 1 (open-label, dose-escalation)	Allogeneic BM-MSCs	Intravenous	Safety confirmed. Well-tolerated. High-dose group showed reduction in motor scores (UPDRS) and peripheral inflammatory markers.	NCT02611167	[205]
MS	Phase 2 (randomized, double-blind, placebo-controlled)	Autologous MSC-neural progenitors	Intrathecal	Mixed efficacy. Primary endpoint not met. Subgroup with higher disability showed improved walking speed and bladder function.	NCT03355365	[207]
MS	Phase 1/2	Allogeneic UC-MSCs and their conditioned media	Intravenous and intrathecal	Safety confirmed. Both single- and two-dose UC-MSC regimens improved disability; two-dose treatment showed greater clinical and anti-inflammatory benefits.	NCT03326505	[208]
ALS	Phase 3 (randomized, double-blind, placebo-controlled)	BM-MSCs	Intrathecal	Safety confirmed. Biomarker improvement. Primary efficacy endpoint not met. Pre-specified subgroup showed functional benefit.	NCT03280056	[210]
ALS	Pilot safety study	BM-MSC-derived EVs	Intravenous	Safety of Secretome. First-in-human evidence that MSC-EVs are safe. 30% of subjects showed stable functional scores over 3 months.	N/A	[211]
HD	Phase 2 (randomized, double-blind, placebo-controlled)	Allogeneic human DPSCs	Intravenous	Met primary safety endpoint. Statistically significant improvement in Total Motor Score and Total Functional Capacity compared to placebo.	NCT03252535	[213]
TBI	Phase 2 (double-blind, randomized, prospective, surgical sham-controlled)	Allogeneic BM-MSCs	Intracranial	Safety confirmed. Motor recovery. Met primary endpoint. Significant and sustained improvement in motor status at 24 and 48 weeks.	NCT02416492	[217]
SCI	Phase 1	Autologous AD-MSCs	Intrathecal	Safe and well-tolerated. 7 out of 10 patients showed improvement in AIS grade at final follow-up.	NCT03308565	[220]
SCI	Phase 1/2 (single-center, open-label, parallel-group RCT)	Autologous BM-MSCs and allogeneic UC-MSCs	Intrathecal/perilesional	Comparable safety. Both autologous BM-MSCs and allogeneic UC-MSCs showed significant improvements in total AIS scores.	NCT04288934	[222]

N/A, not applicable. Abbreviations: AD: Alzheimer’s disease; AD-MSCs: Adipose-derived mesenchymal stem cells; AIS: American Spinal Injury Association Impairment Scale; ALS: Amyotrophic lateral sclerosis; BM-MSCs: Bone marrow mesenchymal stem cells; DPSCs: Dental pulp stem cells; EVs: Extracellular vesicles; HD: Huntington’s disease; MS: Multiple sclerosis; PD: Parkinson’s disease; RCT: Randomized controlled trial; SCI: Spinal cord injury; TBI: Traumatic brain injury; UC-MSCs: Umbilical cord mesenchymal stem cells; UPDRS: Unified Parkinson’s Disease Rating Scale

### Hurdles to clinical application

Despite the promising results from early-stage clinical trials, several significant hurdles must be overcome to facilitate the widespread clinical application of MSC and MSC-derivative therapies.

A major challenge lies in the lack of standardized protocols for the isolation, expansion, characterization, and manufacturing of MSCs and their secretome [223]. MSCs are not a homogenous population, and their properties can vary significantly depending on the tissue source (bone marrow, adipose, umbilical cord, etc.), donor characteristics (e.g., age), and culture conditions (e.g., two-dimensional vs. three-dimensional culture, media components) [224]. This heterogeneity complicates the comparison of results across different studies and poses a regulatory challenge for defining a consistent therapeutic product. Developing robust potency assays to predict in vivo efficacy is critical. For cell-free products like exosomes, challenges in standardization are even more pronounced, with a need for consensus on methods for isolation (e.g., ultracentrifugation vs. chromatography), characterization, and quantification to ensure product quality and consistency. Scalable manufacturing processes that adhere to Good Manufacturing Practice standards are essential for producing the large quantities of cells or exosomes required for clinical use.

Defining the pharmacokinetics and pharmacodynamics of MSCs—specifically the optimal dose, route of administration, and therapeutic window—remains a critical and unresolved issue. Preclinical studies have shown that therapeutic effects can be dose-dependent, with both insufficient and excessively high doses potentially being less effective [168]. The route of administration—intravenous, intra-arterial, intrathecal, or intranasal—influences the biodistribution, CNS penetrance, and safety profile of the therapy, and the optimal route may be disease-specific [225]. The timing of intervention is also crucial, particularly in acute injuries like stroke and TBI, where early administration may be more effective at curbing secondary injury cascades [226]. However, delayed treatment may also be beneficial by targeting later-phase repair processes [196]. Rigorous preclinical and clinical studies are needed to systematically optimize these parameters for each neurological condition.

While MSCs have demonstrated a strong safety profile in numerous trials, with minimal immunogenicity and low risk of adverse events, long-term safety monitoring is still essential. Concerns, though largely theoretical for MSCs, include the potential for thromboembolism, ectopic tissue formation, malignant transformation, and unforeseen immunological reactions. Cell-free therapies using exosomes are considered safer as they eliminate risks associated with live, replicating cells. However, the long-term biological effects of administering large quantities of exosomes, including their potential impact on off-target cells and tissues, require thorough investigation. Addressing these multifaceted challenges through rigorous, well-designed preclinical and clinical research will be paramount to successfully translating the immense promise of MSC-based therapies into standard clinical practice for patients with neurological diseases.

It is also worth noting that a notable disparity exists between the efficacy of MSC therapies in animal models and the variable outcomes observed in clinical trials, a disconnect likely influenced by the limitations of current preclinical models. Species-specific differences in immune physiology play a role; murine and human immune systems exhibit distinct transcriptional responses to trauma and inflammation, suggesting that immunomodulatory mechanisms validated in mice may not always fully recapitulate human responses [227, 228]. In addition to these biological divergences, there is a contrast in host health status. While standard laboratory animals are typically young, genetically identical, and housed in specific pathogen-free conditions, the target clinical population often comprises elderly patients with diverse genetic backgrounds and comorbidities. These factors may independently alter the neuroinflammatory landscape and potentially affect endogenous repair mechanisms. Furthermore, anatomical scaling presents a pharmacokinetic challenge, as the diffusion dynamics and distribution volumes differ significantly between rodent and human brains; consequently, systemic delivery protocols validated in rodents may not achieve comparable therapeutic concentrations at the lesion site in humans [229, 230]. Moreover, the pathology of certain transgenic models may not fully capture the chronic, multifactorial nature of sporadic human neurodegeneration. Collectively, these discrepancies suggest that therapeutic potency could be overestimated in some preclinical studies, indicating that future research might benefit from incorporating more predictive models, such as aged animals with comorbidities or humanized systems.

## Conclusion and future directions

The landscape of therapeutic development for neurological disorders is being reshaped by the significant advances in our understanding of MSCs’ biology and their powerful immunomodulatory capabilities. The recognition that neuroinflammation is a central, unifying driver of pathology across a wide spectrum of acute and chronic CNS diseases has provided a clear and compelling target for intervention. MSC-based therapies, through their sophisticated paracrine mechanisms, have emerged as a uniquely promising strategy to address this target. The paradigm shift from a focus on cell replacement to an appreciation of the MSC secretome—comprising EVs, exosomes, and a rich milieu of soluble factors—has paved the way for safer and more translatable cell-free therapeutic approaches.

This review has synthesized a large body of preclinical evidence demonstrating that MSCs and their derivatives can effectively rebalance the neuroinflammatory microenvironment. They achieve this by orchestrating a phenotypic switch in glial cells, pushing microglia and astrocytes away from their neurotoxic, pro-inflammatory states and towards neuroprotective, pro-regenerative phenotypes. These cellular effects are underpinned by the targeted modulation of critical intracellular signaling pathways, such as the suppression of NF-κB, MAPK, and NLRP3 inflammasome activation. The bioactive cargo of MSC-derived exosomes, particularly specific miRNAs and proteins like TSG-6, has been identified as the key molecular messenger system that executes these complex regulatory functions. The therapeutic potential of this approach has been validated in diverse models of AD, PD, MS, stroke, TBI, and SCI, where MSC-based interventions consistently lead to reduced neuropathology and improved functional outcomes.

Despite this immense promise, the journey from bench to bedside is ongoing and requires dedicated efforts to overcome existing challenges. The future of this field will likely focus on several key areas. First, optimizing and enhancing therapeutic efficacy through advanced strategies will be crucial. Preconditioning MSCs with specific stimuli (e.g., hypoxia, inflammatory cytokines, pharmacological agents) and genetically engineering cells or their exosomes to overexpress specific therapeutic factors (e.g., neurotrophins, anti-inflammatory miRNAs) are powerful methods to create more potent, “next-generation” therapeutics. Second, innovations in delivery systems are vital for maximizing bioavailability and targeting. The continued development of non-invasive intranasal delivery routes and advanced biomaterial scaffolds for sustained local release will be critical for clinical success. Third, rigorous standardization of manufacturing, characterization, and quality control processes is paramount for regulatory approval and widespread clinical adoption. This includes establishing reliable potency assays that can predict the in vivo therapeutic activity of a given MSC or exosome preparation. Finally, well-designed, large-scale, and placebo-controlled clinical trials are essential to definitively establish the efficacy of these therapies in human patients.

In conclusion, MSCs and their secretome represent a new therapeutic platform with the potential to fundamentally alter the treatment of neurological diseases. By targeting the core pathological process of neuroinflammation, these therapies offer a holistic approach that not only protects the nervous system from secondary damage but also fosters an environment conducive to endogenous repair. While significant hurdles remain, the convergence of advances in stem cell biology, bioengineering, and clinical trial design provides a clear and optimistic path forward. Continued interdisciplinary research will be key to unlocking the full potential of MSC-based therapies and bringing this new class of regenerative medicine to patients in need.

## Data Availability

Not applicable.

## Abbreviations

AD: Alzheimer’s disease
AD-MSCs: Adipose-derived mesenchymal stem cells
AIS: American Spinal Injury Association Impairment Scale
ALS: Amyotrophic lateral sclerosis
Aβ: Amyloid-β
APP: Amyloid precursor protein
BBB: Blood-brain barrier
BDNF: Brain-derived neurotrophic factor
BM-MSCs: Bone marrow mesenchymal stem cells
CNS: Central nervous system
DAM: Disease-associated microglia
DAMPs: Damage-associated molecular patterns
EVs: Extracellular vesicles
EAE: Experimental autoimmune encephalomyelitis
GSDMD: Gasdermin D
HD: Huntington’s disease
HMGB1: High mobility group box 1
ICH: Intracerebral hemorrhage
IL-1β: Interleukin-1β
IL-6: Interleukin-6
iNOS: Inducible nitric oxide synthase
lncRNAs: Long non-coding RNAs
LPS: Lipopolysaccharide
MAPK: Mitogen-activated protein kinase
MAPK9: Mitogen-activated protein kinase 9
miRNAs: MicroRNAs
MMPs: Matrix metalloproteinases
MS: Multiple sclerosis
MSC-CM: Mesenchymal stem cell-conditioned medium
MSCs: Mesenchymal stem cells
MyD88: Myeloid differentiation primary response gene 88
NETs: Neutrophil extracellular traps
NF-κB: Nuclear factor-kappa B
NIHSS: National Institutes of Health Stroke Scale
OM-MSCs: Olfactory mucosa MSCs
PD: Parkinson’s disease
PDCD4: Programmed cell death 4
PGE2: Prostaglandin E2
PS1: Presenilin 1
PTX3: Pentraxin 3
ROS: Reactive oxygen species
SAH: Subarachnoid hemorrhage
SCI: Spinal cord injury
sEVs: Small EVs
STAT6: Signal transducer and activator of transcription 6
TBI: Traumatic brain injury
TGF-β: Transforming growth factor-β
Th17: T helper 17
TLR: Toll-like receptor
TNF-α: Tumor necrosis factor-α
Tregs: Regulatory T cells
TREM1: Triggering receptor expressed on myeloid cells 1
TSG-6: Tumor necrosis factor-α-stimulated gene 6
UC-MSCs: Umbilical cord mesenchymal stem cells
